# Effectiveness of protection areas in safeguarding biodiversity and ecosystem services in Tibet Autonomous Region

**DOI:** 10.1038/s41598-021-03653-6

**Published:** 2022-01-21

**Authors:** Kaipeng Xu, Xiahui Wang, Jinnan Wang, Jingjing Wang, Rongfeng Ge, Rensheng Tian, Huixia Chai, Xin Zhang, Le Fu

**Affiliations:** Center of Eco-Environmental Zoning, Chinese Academy of Environmental Planning (CAEP), Beijing, 100012 People’s Republic of China

**Keywords:** Ecology, Ecology, Environmental sciences, Environmental social sciences, Natural hazards

## Abstract

The Tibet Autonomous Region of China constitutes a unique and fragile ecosystem that is increasingly influenced by development and global climate change. To protect biodiversity and ecosystem services in Tibet, the Chinese government established a system of nature reserves at a significant cost; however, the effectiveness of nature reserves at protecting both—biodiversity and ecosystem service functions in Tibet is not clear. To determine the success of existing nature reserves, we determined importance areas for the conservation of mammal, plant, bird, amphibian, and reptile species, and for the protection of ecosystem service functions. The results indicated that important conservation areas for endangered plants were mainly distributed in the southern part of Nyingchi City, and for endangered animals, in the southern part of Nyingchi and Shannan Cities. Extremely important conservation areas for ecosystem service functions of carbon sequestration, water and soil protection, and flood regulation were mainly distributed in the southern part of Nyingchi and Shannan Cities, northern and southeastern parts of Nagqu City, and southern part of Ngari area. Based on an analysis of spatial overlap in protection areas, we conclude that existing natural reserves need to be expanded, and new ones need to be established to better protect biodiversity in Tibet Autonomous Region.

## Introduction

The Tibet Autonomous Region, located within the Qinghai–Tibet Plateau in southwestern China, constitutes a unique and fragile ecosystem^1^. The region is the birthplace of several main rivers of Asia including the Yangtze, Lancang, and others. The region’s water and soil conservation functions are important for water quality and quantity, and the maintenance of agricultural irrigation and other human production activities in the middle and lower reaches^2^. The region’s water and soil conservation functions are important for maintaining water quality and quantity, and maintenance of agricultural irrigation and other human production activities in the middle and lower reaches^3^.

Environmental problems caused by human activities and climate change in the Tibet Plateau have become a serious concern for ecologists and managers^4^. The Chinese government launched an ecological restoration plan with the purpose of slowing down or reversing environmental degradation^5^. Between 1979 and 2015, the investment in restoration activities reached 90 billion yuan. Although vegetation coverage on the Qinghai–Tibet Plateau has increased in response to these activities, there are few reports on the effect of the ecological restoration plan^6^.

However, Tibet faces increasingly pressing climate change problems^7^. A recent study showed that the average temperature in Tibet increased at the rate of 0.32 °C every 10 years from 1961 to 2015; this increase was noticeably higher than the global and the national temperature rise. Climate warming resulted in a reduction of glacier area in the Qinghai–Tibet Plateau, and an increase in melt water volume^8^. Climate change also caused a rise in the lower limit of the plateau permafrost, and strengthening of the freeze–thaw ablation; this led to land desertification, and a breakdown in windbreak, sand stabilization, and water and soil conservation functions of grasslands. Dominant plant species in some areas declined gradually because of global warming, and many weedy plants and toxic herbs appeared, endangering the native biological diversity of the Tibet Region^9^. Therefore, planning for protecting biological diversity and ecosystem service function in Tibet Region is urgent^10^.

The ecosystems of Tibet are considered fragile due to the special geological location, natural conditions, and the relatively low economic development level of the area; however, the variety of ecosystem service functions comprises an important part of ecological security for the region and the country^11^. It has been predicted that biological diversity will increase in the southeastern part of the Qinghai–Tibet Plateau as a result of permafrost thawing; however, as climate becomes increasingly drier, desertification is likely to expend, and biological diversity in the northern part of the Plateau will likely decrease.

The effects of ecological restoration and the impact on ecological environment in Tibet have been extensively researched^12^. However, a great need exists to evaluate the spatial distribution and conservation effectiveness of protected areas in Tibet Autonomous Region^13^. Identification of key areas of ecosystem services and biodiversity will allow the establishment of new targeted ecological restoration areas, and the reduction of environmental degradation^14^. In this study, we focused on areas which protect biodiversity and ensure the security of ecosystem services. Our specific objectives were: (1) to map the spatial distribution of important areas for biological diversity protection in Tibet, (2) determine the spatial pattern of ecosystem services, and (3) analyze protection status and nature reserve system planning.

## Research methods

### Study area

Tibet Autonomous Region is located at the southwest frontier of China, with its geographic location between longitude 78 25′–99 06′ E, and latitude 26° 50′–36° 53′ N; the region borders with the Xinjiang Uygur Autonomous Region and Tibet to the north, Sichuan and Yunnan Provinces to the east, and Nepal, India, Bhutan, and Myanmar to the southeast^15^. It is an important gateway of southwestern China^16^.

Tibet Autonomous Region has an area of 12.02 million square meters, accounting for 1/8 of China’s land area^17^. In 2018, permanent resident population in the region reached 3.36 million. Regional DGP was just RMB 134.56 billion. Compared with central and eastern China, economic development in Tibet is lagging, and it is the least developed of the provinces^18^.

**Figure 1:**
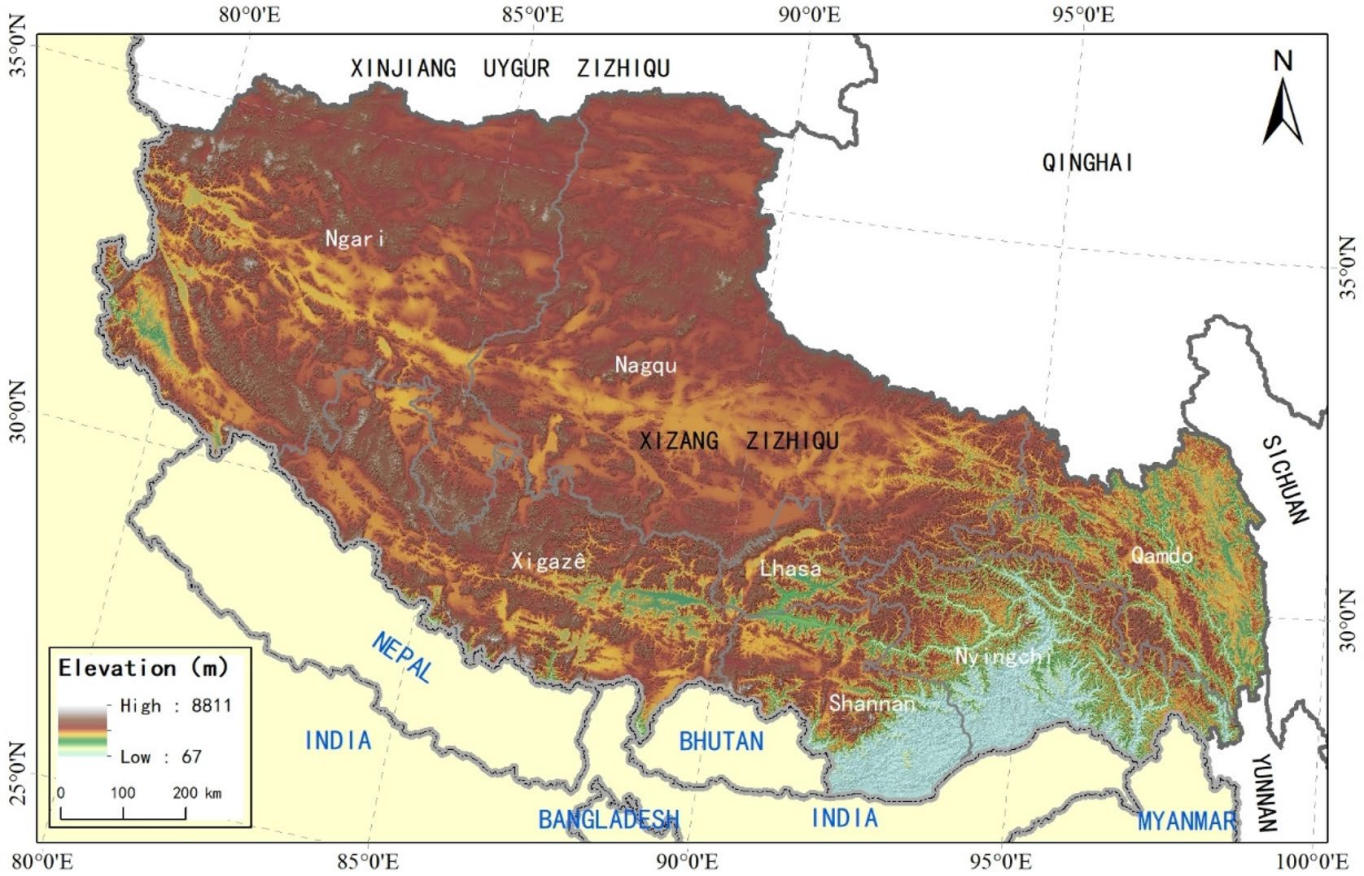
The geographical elevation of the study area and the location map of the surrounding countries and provinces. We created this figure in using ArcGIS 10.5 for maps (URL: http://www.esri.com/).

Average elevation in Tibet Autonomous Region reaches 4728 m, with elevations of > 5000 m spread over 0.467 million square meters, making up 39.8% of the region’s total area mainly in the west; area with elevation ≤ 3000 m accounts for 4.7% of the total area at only 57,000 square meters, and is mainly distributed in the south of Nyingchi City and Shannan City (Fig. 1).

Rainfall in Tibet exhibits strong spatial differences, and is unevenly distributed during the year, with prominent wet and dry seasons. Average temperature in Tibet decreases from southeast to northwest. The plateau area of Tibet experiences strong winds of long duration. The large territory of Tibet has diversified habitat types^19^. It features all terrestrial ecosystem types including peculiarities that other regions of China and other countries in the world do not have. As a unique regional environmental unit, Tibet has given rise to specific biological groups, with many rare wild plants and animals, and is the main differentiation and formation center of world mountain species^20^. Tibet is also a region with rich biodiversity specific to high altitudes, forms a natural germplasm bank of alpine organisms, and contributes to global biodiversity protection.

### Data sources

To determine land cover/land use, we used land coverage data from global land coverage product (http://www.globallandcover.com/) developed by the State Geological Information Center. The images used for classification were mainly 30 m multi-spectral images, including LandsatTM 5 ETM+ multi-spectral image and multi-spectral image of China Environmental Disaster Reduction Satellite (HJ-1)^21^. This series of product raises spatial resolution an order of magnitude, with the overall classification precision reaching 80%. Vegetation coverage data were obtained from Moody Image with an inversion based on a pixel binary model. The calculation of vegetation coverage was based on MODIS data products provided by DISC, NDVI data (MOD13Q1) of NASA. We adopted a remote sensing estimation method for the calculation of vegetation coverage, based on pixel binary model and NDVI values as parameters^22^.

Plant distribution information was obtained from the China Plants Database; spatial distribution data of mammals, amphibians, and reptiles were obtained from IUCN, and Colored Atlas of Chinese Amphibians and Their Distributions, supplemented with China’s Mammal Diversity and Geographical Distribution; bird spatial distribution data originated from Bird Life International; elevation and habitat vegetation types from China Plants Database, IUCN Red List, Bird Life International, and recent studies^23^. Elevation data came from the 90 m digital elevation model obtained in radar terrain detection mission of the NASA shuttle.

Biomass was calculated with the biomass method and data accumulation NNP method of above ground biomass. Vegetation index-biomass is an empirical statistical model based on field measured forest shrub biomass data and quickbird, and then reverse-based on quickbird from biomass; cumulative NNP method was confirmed with the growth period (start and end periods) of grassland or field, cumulative NPP for a growth period was calculated for above ground biomass and CASA model adopted an NPP algorithm^24^. Natural reserve data were obtained from the China Ministry of Ecology and Environment.

We calculated other ecosystem functions, including carbon fixation, water and soil conservation, windbreak and sand stabilization, and flood regulation using 5-year (2010–2015) evaluation project of the Chinese ecosystem environment^25^. Details of calculations are given below.

### Mapping of biological diversity

To map the important areas of biodiversity conservation, we selected threatened species from the IUCN Red List or Chinese Red List indicator species, including species in critically endangered (CR), endangered (EN), and vulnerable (VU) categories^26^. The final selected list contained a total of 1534 species, including 955 plants, 152 mammals, 127 birds, 177 amphibians, and 123 reptiles^27^. Distribution information for plants was obtained from the Scientific Database of China Plant Species. Range maps for mammals, amphibians, and reptiles were obtained from the IUCN. Range maps for birds were acquired from BirdLife International. We refined the potential habitat for each species based on specific distribution area, elevational range, and vegetation. Data on specific distribution areas were obtained from the Institute of Zoology, Chinese Academy of Sciences; habitat requirements for elevation and vegetation for each species were derived from the Scientific Database of China Plant Species, the IUCN Red List, BirdLife International, and recent studies^28^.

Important areas for species conservation were identified by summing up weighted potential habitats for each taxon. To describe the relative importance of different IUCN categories, we gave weights of 3, 2, and 1 to categories CR, EN, and VU, respectively. For each taxon, we normalized the summed values separately to the range of 0–100 using the minimum–maximum normalization method, with 100 being the most important and 0 being the least important^29^.

Spatial overlapping was conducted to obtain the importance value of biodiversity protection for the 5 groups of species, and the maximum value was the total importance value of biological diversity protection for all species together. Based on the importance value, the top cumulative 20% area was taken as extremely important for biological diversity^30^.

### Mapping of ecosystem services

We considered five key regulating ecosystem services: water retention, soil retention, sandstorm prevention, carbon sequestration, including both biophysical supply and supply weighted by the number of people benefiting^31^. Data for these calculations were obtained from the National Ecosystem Assessment Project 2000–2015^32^.

A. We calculated carbon sequestration as the difference between net primary productivity and soil respiration, as follows^33^:
  $${\text{NEP}} = {\text{NPP}} - {\text{RS}},$$
  where NEP, net ecosystem productivity, was carbon sequestration (g C/m^2^/year), NPP was net primary productivity (g C/m^2^/year) of the ecosystem, and RS was carbon release (g C/m^2^) from soil through respiration.
B. Water conservation amount, rainfall, evapotranspiration, and surface runoff are closely related to vegetation type and other factors, and may be calculated using the water balance equation as follows^34^:
  $$TQ = \sum\limits_{i = 1}^{j} {(P_{i} - R_{i} - ET_{i} )} \cdot A_{i},$$
  where *TQ* was the total water conservation amount (m^3^).
  *P*_*i*_ was rainfall (mm); *R*_*i*_was rainstorm runoff (mm), *ETi* was evapotranspiration (mm), *Ai* was the area of ecosystem type *i*; *i* was ecosystem type in the study area; *j* was the total number of ecosystems in the study area.
C. To calculate soil conservation, we used the general soil loss equation (USLE), as modified by.
  $$SC = R \cdot K \cdot LS \cdot \left( {1 - C} \right),$$
  where SC was the soil conservation amount (t hm^−2^ a^−1^); R was rainfall erosivity factor (MJ mm hm^−2^ h^−1^ a^−1^); K was soil erodibility factor (t hm^2^ h hm^−2^MJ^−1^ mm^−1^); LS was terrain factor; C was vegetation coverage factor^35^.
D. To calculate sand stabilization amounts, we used the revised wind erosion equation, which incorporates effects of wind speed, rainfall, temperature, soil texture, terrain, and vegetation coverage on soil erosion and soil conservation^36^.
  $${\text{SR = S}}_{{{\text{Lpotential}}}} - S_{L}$$
  $$R_{k} = {\raise0.7ex\hbox{${{\text{SR}}}$} \!\mathord{\left/ {\vphantom {{{\text{SR}}} {{\text{S}}_{{{\text{Lpotential}}}} }}}\right.\kern-\nulldelimiterspace} \!\lower0.7ex\hbox{${{\text{S}}_{{{\text{Lpotential}}}} }$}}$$
  where, SR was the amount of sand stabilization (t km^−2^ a^−1^), R_k_ was sand stabilization rate, *SL*_*potential*_ was the potential wind erosion amount, and *S*_*L*_ was the actual soil erosion amount (t km^−2^ a^−1^).
E. Flood regulation capacity of lakes was based on the relationship between the adjustable water storage capacity of different lakes and lake surface area; the adjustable water storage capacity of the lakes was further estimated based on lake surface area^37^.
  $$Ln\left( {C_{r} } \right) = 0.{678}\,Ln\left( A \right) + {6}.{636} \, (N = {6}, R^{{2}} = {\text{ }}0.{963}),$$
  where *C*_*r*_ was adjustable water storage capacity (10,000 m^3^); A was lake surface area (km^2^).
  $$C_{r} = 0.{35}\,C_{t} \, (N = {46}0, \,R^{2} = 0.{81}0),$$
  where *C*_*r*_ was flood control storage (10,000 m^3^), *C*_*t*_was total storage capacity.

Flood storage capacity of marshland takes into account surface water retention in marshland, calculated for the average submerged 1 m in flood period and marshland area^38^.

Similar to the importance index for endangered species, we normalized the biophysical supply value into the importance index value ranging from 0 to 100 using the minimum–maximum normalization method. We also defined an important area for ecosystem service provision as the top 20% cumulative area according to the importance index value. The overall importance index map for overall ecosystems service was the maximum value of the four services layers.

### Effects of ecological protection

We used spatial overlapping to analyze the protection effect of natural reserves on biodiversity and ecosystem service function^39^. If the coverage of key habitats in nature reserves was higher than that of nature reserves in Tibet Autonomous Region, we defined it as good protection reserves effect on threatened species by protected areas network^40^. For ecosystem services, if the natural protection zones of biophysical supply (or of the weighted number benefiting from the supply) relative to its total supply were greater than the area of Tibet autonomous region, and land supply of biophysical supply (or the weighted number benefiting from the supply), we defined it as a better protective effect on the service function of the reserve network, or as poorly represented in terms of threatened species or ecosystem services.

### Protection gap analysis

We analyzed the need for establishing new nature reserves or expanding existing ones to better cover high priority areas for biodiversity and ecosystem services. Priority areas were delimited by using the top 20% of habitat for biodiversity conservation and for ecosystem service provision according to the importance index value. Protection net is a design based on important area protection for both biological diversity and ecological system service function^41^. Protection gap analysis quantifies the need for establishment of new, or expansion of existing protection sites; an overlap analysis of the distribution of areas of importance for biological diversity protection, ecosystem service protection, and that of current nature reserves. then by recognizing the spatial unit without the distribution of important area of biological diversity and ecosystem service protection^42^.

## Results

### Spatial distribution of important areas for biological diversity protection

We assigned species to the following categories: extremely endangered, endangered, and vulnerable, based on IUCN and China Species Red List for the Tibet Autonomous Region. In total, 202 species were selected as important protection species; among them were 105 plant, 46 mammal, 23 bird, 16 amphibian, and 12 reptile species.

**Figure 2:**
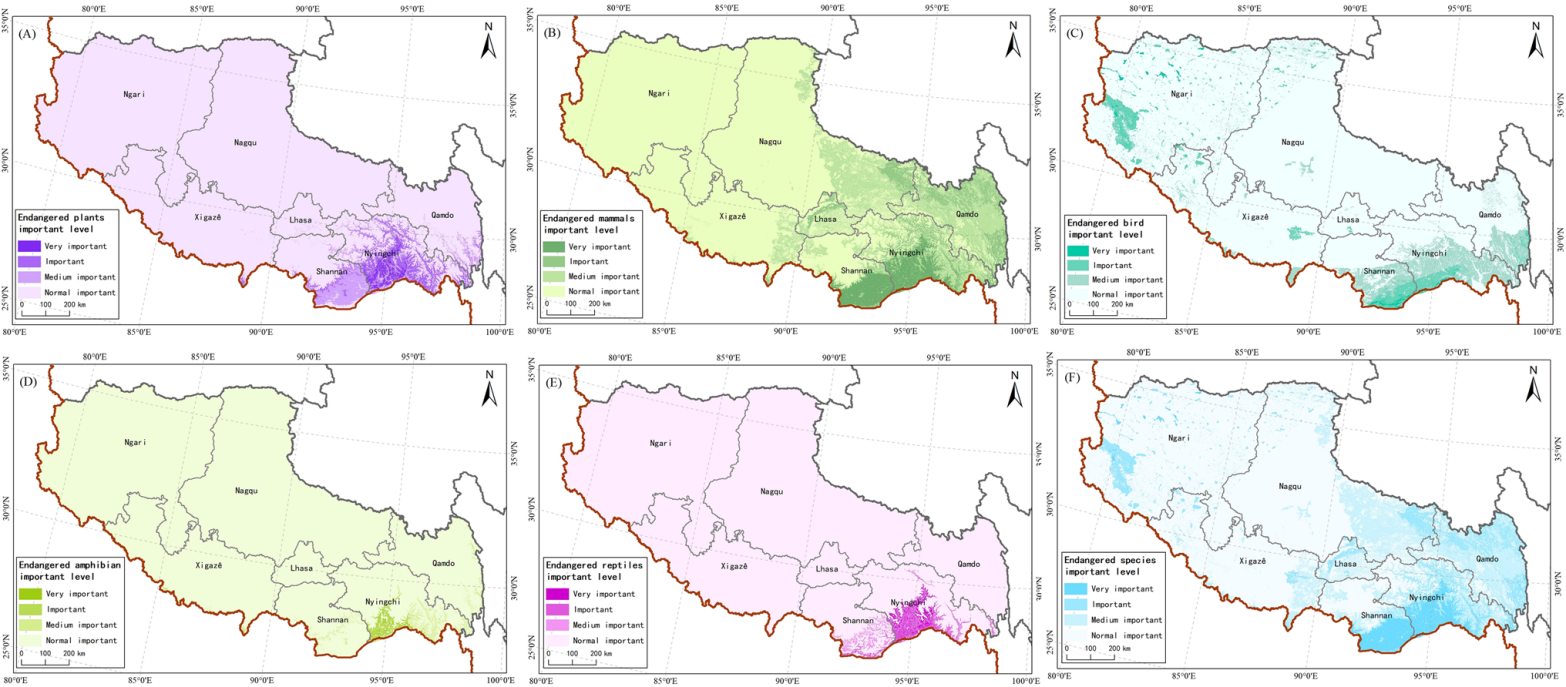
Spatial pattern of Biodiversity importance of China’s Tibet autonomous region, (**A**) distribution of the importance of endangered plants, (**B**) distribution of importance of endangered mammals, (**C**) distribution of importance of endangered birds, (**D**) distribution of importance of endangered amphibians, (**E**) distribution of importance of endangered reptiles, (**F**) distribution of importance of endangered species. We created this figure in using ArcGIS 10.5 for maps (URL: http://www.esri.com/).

Important areas for protection of endangered plants in Tibet Autonomous Region were mainly distributed in the southern part of Nyingchi City (Fig. 2A). In that, extremely important and important areas totaled respectively 6383.31 and 36,052.50 km^2^ accounting for 0.53 and 3.00% of the total area of Tibet (Table 1).

**Table 1:** Area (km^2^), and its proportion (%) to total area with different levels of importance for biodiversity conservation in Tibet.

Biodiversity type	Extremely important		Important		Medium importance		General importance	
	Area	%	Area	%	Area	%	Area	%
Plants	6383.31	0.53	36,052.50	3.00	44,163.94	3.67	1,115,359.50	92.80
Mammals	50,365.63	4.19	65,749.56	5.47	187,642.38	15.61	898,201.69	74.73
Birds	6674.69	0.56	52,993.25	4.41	87,663.19	7.29	1,054,628.13	87.74
Amphibians	2336.75	0.19	7164.81	0.60	12,731.81	1.06	1,179,725.88	98.15
Reptiles	6854.81	0.57	16,851.94	1.40	10,445.75	0.87	1,167,806.75	97.16
All	54,994.25	4.58	102,822.25	8.55	197,048.69	16.39	847,094.06	70.48

Important areas for protection of endangered mammals were mainly distributed in the southern part of Nyingchi and Shannan Cities (Fig. 2B). In that, extremely important and important areas totaled respectively 50,365.63 and 65,749.56 km^2^, accounting for 4.19 and 5.47% of the total area of Tibet (Table 1).

Important areas for protection of endangered birds in Tibet were mainly distributed in the south of Nyingchi and Shannan Cities and in the west of Ngari Region (Fig. 2C). In that, extremely important and important areas totaled respectively 6674.69 and 52,993.25 km^2^, accounting for 0.56 and 4.41% of the total area (Table 1).

Important areas for protection of amphibians in Tibet were mainly found in the south of Nyingchi City (Fig. 2D); extremely important and important areas totaled 2336.75 and 7164.81 km^2^, respectively, accounting for 0.19 and 0.60% of the total area of Tibet.

Important areas for reptile protection were mainly distributed in the south of Nyingchi and Shannan Cities (Fig. 2E). In that, extremely important and important areas totaled 6854.81 and 16,851.94 km^2^, respectively, accounting for 0.57 and 1.40% of the total area of Tibet (Table 1). Important areas for protection of all endangered species in Tibet Autonomous Region were mainly distributed in the south of Nyingchi and Shannan Cities, and in the west of Ngari Region (Fig. 2F). In that, extremely important and important areas totaled 54,994.25 and 102,822.25 km^2^, respectively, accounting for 4.58 and 8.55% of the total area of Tibet (Table 1).

### Importance pattern of ecosystem service function

**Figure 3:**
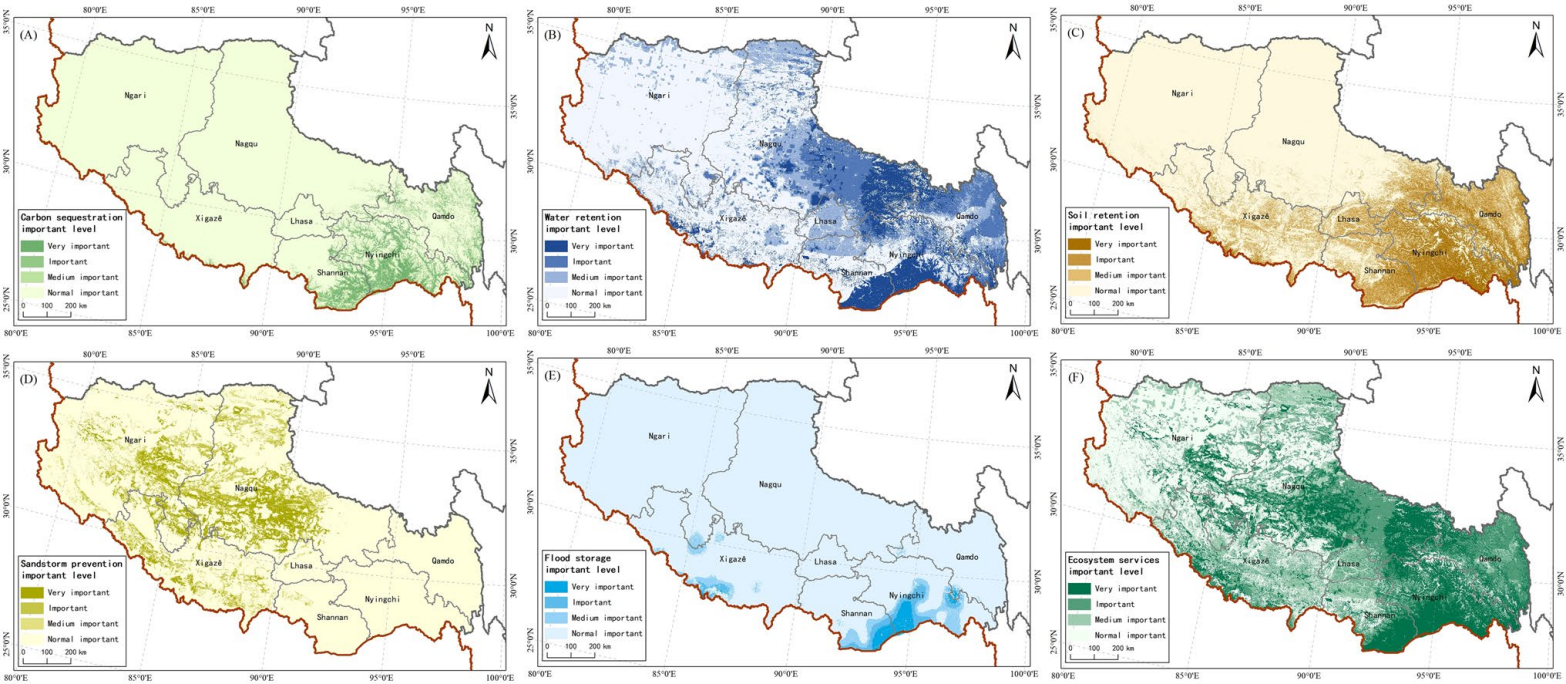
Spatial distribution pattern of ecosystem services importance in Tibet, (**A**) spatial distribution of the importance of carbon sequestration, (**B**) spatial distribution of the importance of water retention, (**C**) spatial distribution of the importance of soil retention, (**D**) spatial distribution of the importance of sandstorm prevention, (**E**) spatial distribution of the importance of flood storage, (**F**) spatial distribution of the importance of ecosystem services. We created this figure in using ArcGIS 10.5 for maps (URL: http://www.esri.com/).

For biophysical supply, areas that were extremely important and important for carbon sequestration were mainly distributed in the south of Nyingchi and Shannan Cities, and on the periphery of Qamdo City; these areas accounted for 3.52 and 2.99%, respectively, of the total area of Tibet (Table 2). Areas that had medium importance for carbon sequestration were mainly distributed in the south of Qamdo City, accounting for 2.77% of the total area (Fig. 3A).

**Table 2:** Area and proportion with different levels of importance for ecosystem services in Tibet.

Type	Extremely important		Important		Medium importance		General importance	
	Area/km^2^	%	Area/km^2^	%	Area/km^2^	%	Area/km^2^	%
Carbon sequestration	42,345.69	3.52	35,773.75	2.98	33,273.63	2.77	1,090,566.19	90.73
Water conservation	143,057.00	11.90	172,578.81	14.36	174,306.44	14.50	712,017.00	59.24
Soil conservation	95,311.19	7.93	105,816.94	8.80	127,116.00	10.58	873,715.13	72.69
Windbreak and sand fixation	79,077.25	6.58	75,374.50	6.27	70,415.75	5.86	977,091.75	81.29
Flood regulation	16,111.69	1.34	20,472.75	1.70	38,616.44	3.21	1,126,758.38	93.74
All	290,653.25	24.18	249,118.50	20.73	224,450.25	18.67	437,737.25	36.42

Extremely important and important areas for water conservation were mainly distributed in the south of Nyingchi and Shannan Cities, and in the southeastern part of Nagqu City, accounting for 11.90% of the total area of Tibet (Table 2). Important areas for water conservation were mainly east of Qamdo and Nagqu Cities, accounting for 14.36% of the total area. Medium importance areas were mainly in the north of Lhasa and Shanshan Cities, accounting for 14.50% of the total area of Tibet (Fig. 3B).

Extremely important and important areas for soil conservation were mainly distributed south of Nyingchi and Shannan Cities, and east of Qamdo City, accounting respectively for 7.93 and 8.80% of the total area of Tibet (Table 2). Medium importance areas for soil conservation were mainly distributed in Xigazê City, accounting for 10.58% of the total area (Fig. 3C).

Extremely important and important areas for sandstorm prevention were distributed in Nagqu City and Ngari Region (Fig. 3D). Extremely important and important areas for flood regulation in Tibet were mainly distributed in the south of Nyingchi and Shannan Cities (Fig. 3E), accounting for 1.34 and 1.70%, respectively (Table 2).

Extremely important areas for ecosystem service protection were mainly distributed south of Nyingchi and Shannan Cities, northwest and southeast of Nagqu City, and south of Ngari Region, accounting for 42.42% of the total area (Table 2). Important areas were mainly distributed in the middle of Qamdo and Nagqu Cities, accounting for 20.73% of the total area. Medium importance areas were mainly distributed in Lhasa and Xigazê Cities, and northwest of Shannan City, accounting for 18.16% of the total area of Tibet (Fig. 3F).

### Comprehensive spatial pattern of ecosystem service

**Figure 4:**
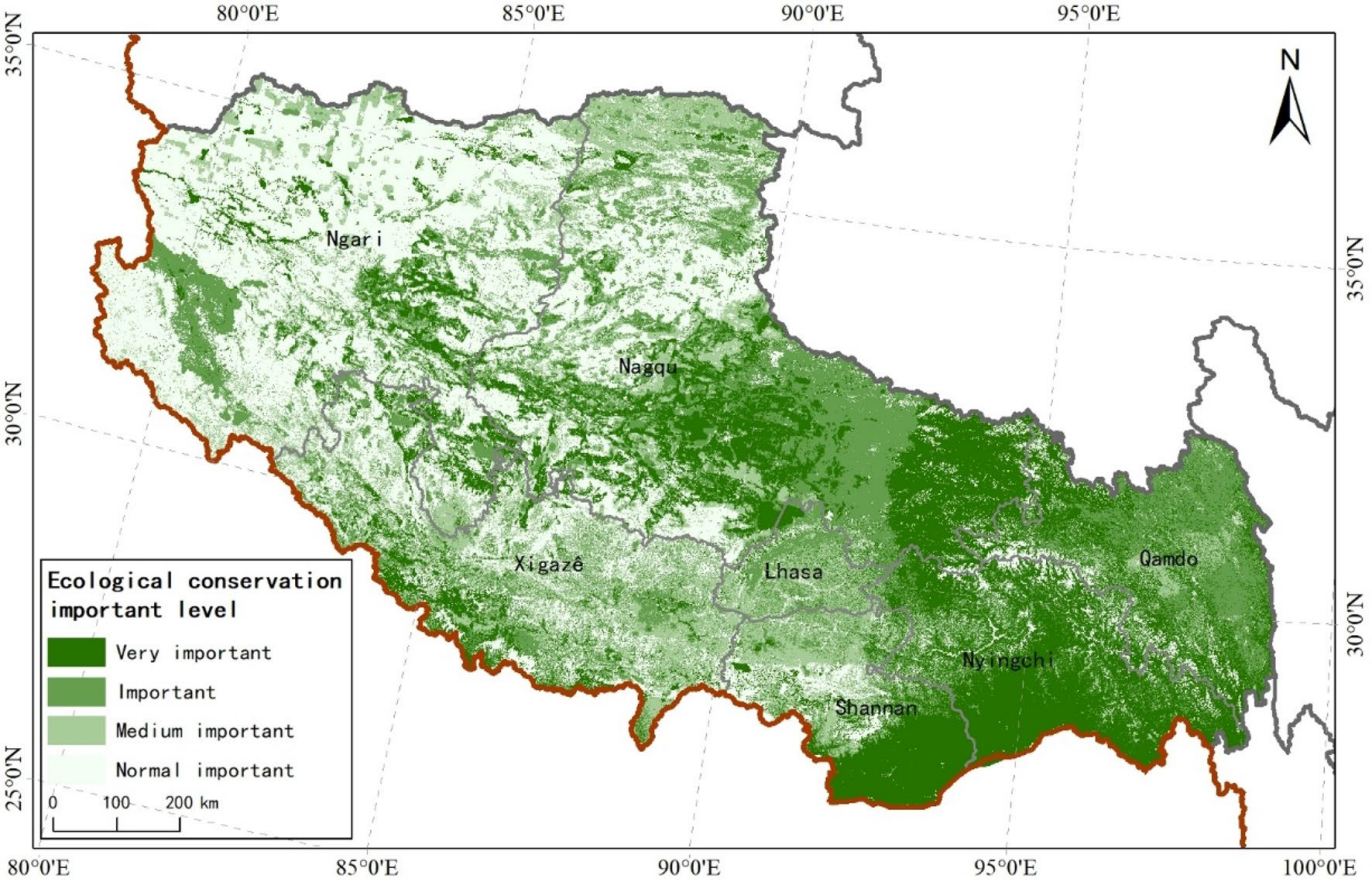
Spatial distribution pattern of ecosystem services in Tibet. We created this figure in using ArcGIS 10.5 for maps (URL: http://www.esri.com/).

Ecological diversity in Tibet Autonomous Region overlapped with areas important for carbon sequestration, water conservation, soil conservation, and flood regulation function; these areas were mainly distributed in the south of Nyingchi City and Shannan City (Fig. 4). Considering both biological diversity and the importance of ecosystem service function, extremely important and important areas of Tibet’s ecological protection totaled 300,024.13 and 281,246.56 km^2^, respectively, accounting for 24.96 and 23.40% of the total area of Tibet; these areas were mainly distributed in the south of Nyingchi City, Qamdo City, Nagqu City, and Shanna City, and in the southeast of Ngari Region. Areas of medium importance for ecological protection totaled 222,343.94 km^2^, accounting for 18.5% of the total area; this was mainly distributed in Xigazê City, Lhasa City, Shannan City, and in the northwest of Nagqu City (Fig. 4).

### Protection status analysis and nature reserve system planning

**Figure 5:**
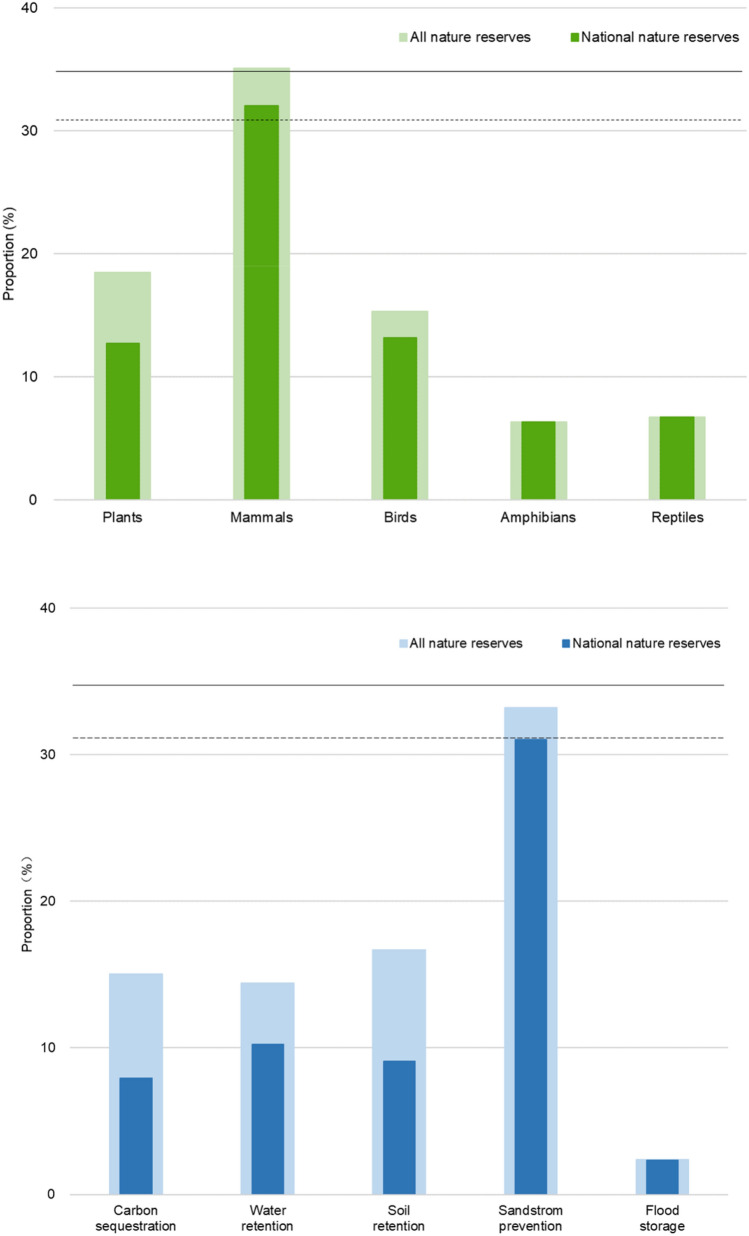
Biodiversity and ecosystem services conservation effectiveness map, solid line indicates the percentage of Tibet area taken up by all nature reserves, dashed line solid line indicated the percentage of Tibet area taken up by national nature reserves.

**Figure 6:**
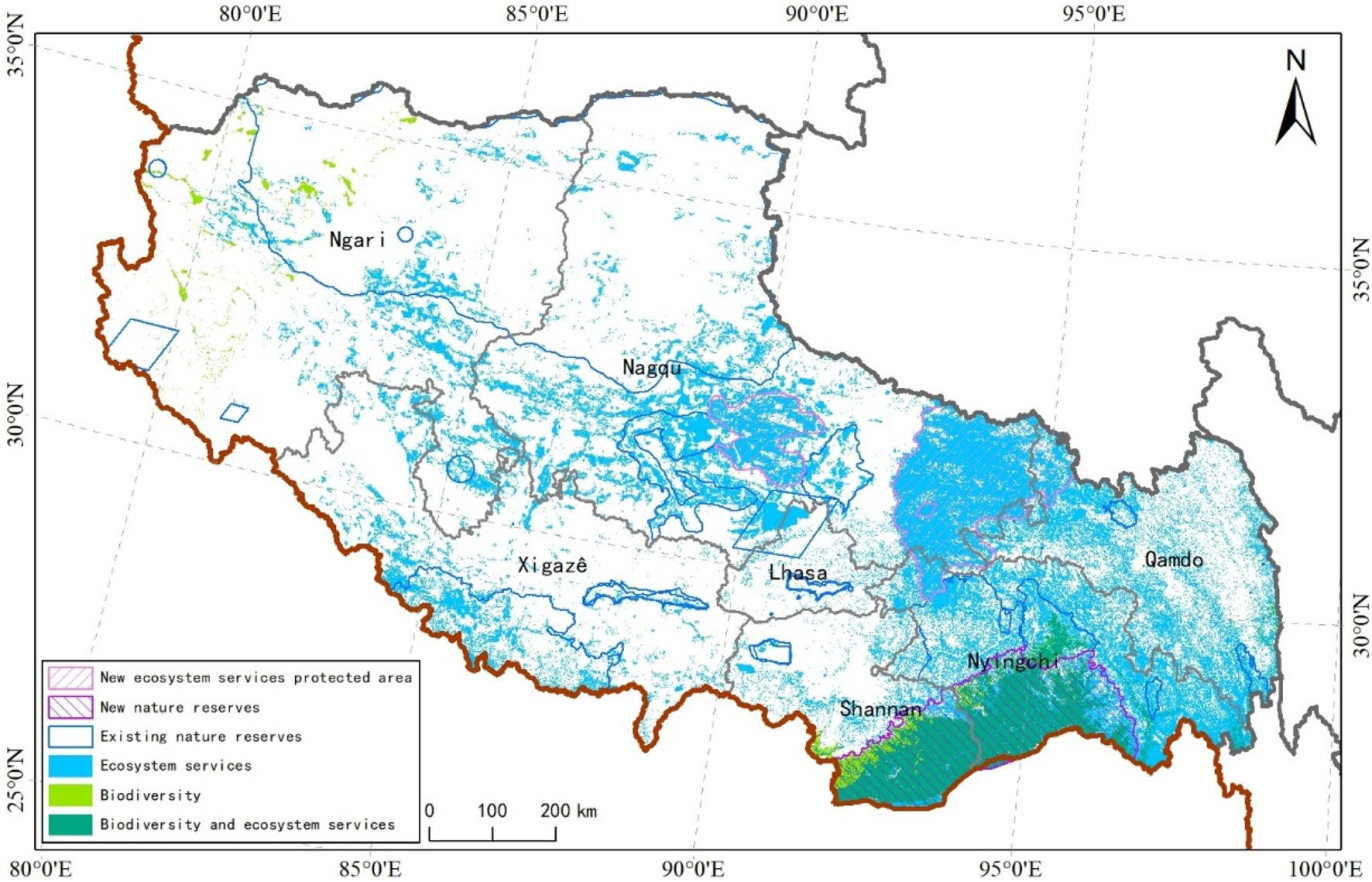
The system planning of Tibet Protected area based on the spatial pattern of biodiversity and ecosystem services. We created this figure in using ArcGIS 10.5 for maps (URL: http://www.esri.com/).

The results of spatial overlapping of nature reserves with different groups of endangered species and ecosystem service function indicated that the existing nature reserves had low effectiveness in biological diversity protection in Tibet Autonomous Region (Fig. 5). National nature reserves are mainly nature reserves for plants, reptiles, mammals, and amphibians, and serve as protection areas for birds, reptiles, and plants more so than for the other endangered species. The effect of all nature reserves on 5 ecosystem services functions used in this study indicating that current nature reserves will not secure ecological functions. In that, however, the protection effect of windbreak and sand stabilization was greater than that of other ecosystem service functions. National nature reserves had a better effect than on windbreak and sand stabilization. Additionally, their effect on water conservation and soil conservation was better than on other ecosystem services. Finally, we have redefined the protected areas system in China's Tibet Autonomous Region (Fig. 6).

## Discussion

### Biological diversity

We found that important and medium-importance areas for endangered species protection in Tibet Autonomous Region were not adjacent to each other. There are two possible reasons for the spatial separation^43^. First, the influence of human activities on biological diversity in Tibet Region played a very important role. Second, targeted protection strategies should be adopted for different protection goals and types of protected areas^44^. For example, we propose that new nature reserves be established or existing ones expanded in the southern part of the Tibet Autonomous Region, with biological diversity as the main protection goal; further, development activities threatening biological diversity should be prohibited or controlled, and the connectedness of plant and animal habitats should be strengthened by constructing ecological corridors, and by restoring habitats^45^. We propose that ecosystem services become the main protection goal in Nagqu Region; there, new ecological protection areas should be established, water conservation ability improved, water and soil loss limited via ecological restoration, and a ban implemented on any new construction that is irrelevant to water protection, windbreak, sand stabilization, and soil conservation^46^.

### Comprehensive spatial pattern

Increasingly more people hope that conservation areas can achieve dual goals—protection of biodiversity and security of ecosystem services. When the importance of biological diversity and ecosystem service function was considered together, we found that the extremely important and important areas for ecological protection accounted for 24.96 and 23.40% of the total area of Tibet. Further, we found that the newly established nature reserves aimed at biological diversity protection and ecosystem service function, and newly established ecological function protection areas aimed at ecosystem service function protection^47^. The area of newly established nature reserves was 60,000 square meters, accounting for 5.05% of the total area of Tibet alone; it was capable of providing important protection for biological diversity^48^. The area of newly established ecological function protection areas reached 65,200 square meters, accounting for 5.42% of the total area of Tibet; this was capable of providing extremely important water conservation, windbreak and sand stabilization, and soil conservation function^49^.

Our research focus ranged from biodiversity to ecosystem services for human well-being. A new reserve can create harmony between man and nature, and improve the health of both. We propose to establish nature reserves in which human activity is highly restricted and a new category of ecosystem service reserves in which those human activities would be allowed that do not affect critical services^50^. We also propose to create a new category of effectiveness appraisal systems in China, which can continuously help to achieve ecosystem services and sustainable development goals. Diversity can also be protected from the micro community level, for example, through community participation^51^, however, that extends beyond the scope of this study.

### Policy significance

Biological diversity and ecosystem service are the two main goals of ecological protection^52^. Nature reserve protection system, which addresses both biological diversity and ecosystem service, is beneficial for all-inclusive interests in protection areas, acceptable for local governments and citizens, and favorable to the construction of nature reserves^53^. However, areas of importance for biological diversity protection in Tibet did not overlap spatially with those of importance for ecosystem service protection; therefore nature reserves in northwestern part of Tibet, with biological diversity as the main protection aim, do not address the protection needs of ecosystem service functions^54^. To protect both—biological diversity and ecosystem service function, current protection system needs to be optimized^55^. In this study, we found overlapping protection areas of biological diversity and ecosystem service mainly in the south of Shannan City and Nyingchi City, with important areas of biological diversity protection relatively small, while those of ecosystem service protection relatively large^56^.

## Conclusions

Our results showed that important areas for endangered plants were distributed mainly south of Nyingchi City. Important areas for endangered mammal protection were found mainly south of Nyingchi and Shannan City. Further, important areas of biological diversity overlapped with those of carbon sequestration, water conservation, soil conservation, and flood regulation to some extent. We conclude that the southern part of Nyingchi City, Qamdo City, Nagqu City, Shannan City, and the southeastern part of Ngari Region are key areas for ecological restoration because of their role in biodiversity and ecosystem services.

This study shed some light on possible goals for ecosystem restoration in the Tibet Region of China; namely, ecosystem restoration should aim for ecosystem services and biological diversity. The study also showcased the insufficient effectiveness of nature reserves and national nature reserves in biodiversity protection in Tibet Autonomous Region, and relatively satisfactory effect on bird, reptile, and plant protection.
